# Proceedings Seventh District Dental Society, Cincinnati, Sept. 17, 1889

**Published:** 1889-11

**Authors:** 


### PROCEEDINGS.

### Minutes of the Seventh District Dental Society, Lincoln Club Hall, Cincinnati, Ohio, Sept. 17th, 1889.

Society met pursuant to adjournment. The President, Dr. E. G. Betty, called the meeting to order at 10 A. M. Minutes of last session read and approved. The following named applicants were elected to active membership:

Drs. J. R. Callahan, R. E. Wyatt, W. D. Phillips, E. P. Binford, A. M. Kempton, C. M. Wright, H. A. Smith. Moved by Dr. J. Taft that delegates be appointed to represent this society in the Ohio State Dental Society. After discussion it was decided to refer the matter to the Executive Committee.

On motion of Dr. Taft, a committee of three was appointed to consider and report to the society the advisability of securing a roster of the practicing dentists of the Seventh District. Pres. appointed on the above committee, Drs. Taft, Sillito and Custer.

Prof. C. M. Wright then read a paper, subject, "Has a Dentist the Legal or Moral Right to Practice General Medicine." Paper was discussed by Drs. Smith, Taft and others.

On motion of Dr. J. R. Callahan, all papers read before this society be referred to the Executive Committee. Adjourned to meet at 2 P. M.

AFTERNOON SESSION.

Society called to order by President at 2:15 P. M. Minutes of morning session read and after corrections approved.

On motion of Dr. Taft, the Secretary was instructed to furnish a report of this meeting to the dental journals for publication.

The following named applicants were elected to active membership: Drs. W. H. H. Hunter, W. D. Kempton, Frank A. Hunter.

The Committee on Delegates to the Ohio State Dental Society presented the following resolution:

*Resolved*, That four delegates to the Ohio State Dental Society be appointed to represent this society and that the following persons be appointed: Drs. B. F. Johnson, P. S. Ballinger, W. D. Kempton, E. P. Binford. Resolution adopted.

Committee on Roster offered the following resolution :

*Resolved,* That the Executive Committee be instructed to procure a complete list of the dentists in the 7th District of the Ohio State Dental Society. Resolution adopted.

Prof. Taft then read a paper, subject, "Clinical Instruction." Paper was discussed by Profs. Smith, Wright, Hunter and others.

A paper was then read by Dr. J. R. Callahan, subject, "Care of Children's Teeth." Paper was discussed by Drs. Kempton, Smith, Taft and others.

Drs. Smith and Betty then exhibited new instruments and other things of interest.

Bill of E. G. Betty for printing, $2.50, ordered paid.

On motion a vote of thanks was tendered the Lincoln Club for the use of the hall. On motion of Dr. Sillito, the society proceeded to ballot to decide on place of next meeting.

The city of Hamilton was selected on first ballot. Time, 3rd Tuesday of May, 1890. Adjourned.

J. R. Callahan, Sec. *pro tem.*

---

Resection of the Inferior Maxilla.—Dr. R. del Castillo-Quartiellerz, has devised a plan for excising the inferior maxilla, which he thinks is a great improvement on the ordinary methods, both from a surgical and from an æsthetic point of view. The method consists essentially in passing a specially constructed trochar through the skin behind the ramus into the mouth, and then making use of the canula as a guide for a chain saw, by means of which the excision is carried out. This procedure, has, of course, to be repeated on the opposite side. The only wounds of the skin are two small openings made by the trochar, the cicatrices of which are small and do not disfigure the patient; there is very little hemorrhage, and there being but a comparatively small wound, the amount of suppuration and consequently the risk of awakening the patient is inconsiderable as compared with that occurring in more severe operations.—*Lancet.*

